# Transmission Line Fault Type Identification Based on Polar Lights Optimizer-Selected Features and a Gramian Angular Field Attention Fusion Network

**DOI:** 10.3390/s26144502

**Published:** 2026-07-15

**Authors:** Guangyi Luo, Tao Mao, Weizhong Ni, Jian Le

**Affiliations:** 1 School of Electrical Engineering and Automation, Wuhan University, Wuhan 430072, China; 2020302192235@whu.edu.cn; 2 School of Mechanical and Electrical Engineering, Wuhan Donghu University, Wuhan 430212, China; maotao@wdu.edu.cn; 3 State Grid Zhejiang Electric Power Co., Ltd. Hangzhou Power Supply Company, Hangzhou 311225, China; niweizhong@hz.zj.sgcc.com.cn

**Keywords:** transmission line fault identification, traveling-wave signal, Gramian Angular Field, polar lights optimizer, feature selection

## Abstract

**Highlights:**

**What are the main findings?**

- A fault type identification framework is proposed by integrating Polar Lights Optimizer-selected multi-domain explicit features with dual-branch Gramian Angular Field image representations.
- Under the representative stratified 8:2 hold-out test, the proposed GAF-PCNN-AT model achieves an accuracy of 95.40% and an average AUC of 0.9900 on six-class transmission line fault identification using historical traveling-wave data.

**What are the implications of the main findings?**

- The proposed method improves the utilization of sensor-acquired traveling-wave signals by jointly exploiting global correlation structures, local difference patterns, and physically interpretable explicit features.
- The framework provides a potential intelligent diagnosis tool for online transmission line monitoring and fault type recognition under imbalanced fault sample conditions.

**Abstract:**

To address class imbalance in transmission line fault traveling-wave samples, the strong non-stationarity of transient traveling-wave features, and the limited identification capability of single-representation methods, this paper proposes a fault type identification method that integrates Polar Lights Optimizer (PLO)-based feature selection with a Gramian Angular Field (GAF) attention fusion network. First, the Borderline Synthetic Minority Over-sampling Technique (Borderline-SMOTE) is applied to balance six fault categories in the training set, and time domain, frequency domain, time–frequency domain, and waveform-edge features are extracted from traveling-wave signals acquired by online monitoring devices. Then, PLO is used to select key explicit features, while the preprocessed traveling-wave sequences are encoded into dual-branch images using the Gramian Angular Summation Field (GASF) and the Gramian Angular Difference Field (GADF). Finally, a Gramian Angular Field–Parallel Convolutional Neural Network–Attention (GAF-PCNN-AT) model is constructed to fuse deep image features with selected explicit features for fault identification. Validation on the independent real test set under a representative stratified 8:2 split shows that the proposed method achieves an accuracy of 95.40% and an average area under the curve (AUC) of 0.9900 in the six-class fault identification task. The results indicate that the proposed method can effectively integrate deep image features of traveling-wave signals with PLO-selected explicit features, thereby providing high identification accuracy and good overall classification performance.

## 1. Introduction

Transmission lines are essential for the secure and stable operation of power systems, undertaking long-distance and large-capacity power transmission. Because transmission lines are exposed to complex outdoor environments for long periods, they are vulnerable to lightning strikes, icing, strong winds, external damage, bird-related incidents, and other factors. As a result, transmission line faults are often characterized by complex causes, short transient durations, subtle waveform differences, and overlapping class boundaries. In recent years, deep learning and machine learning methods have been widely applied to transmission line fault detection, classification, and localization, showing strong capabilities in feature learning and nonlinear mapping [1,2]. However, in practical engineering scenarios, several challenges remain, including unstable fault sample quality, imbalanced class distributions, limited model generalization, and insufficient recognition stability under complex operating conditions.

Transient traveling-wave signals contain high-frequency disturbance information at the initial stage of a fault and can reflect wavefront abrupt changes, energy distributions, and local oscillation characteristics. Existing studies have investigated transmission line fault identification from the perspectives of measured traveling-wave pre-classification, time-domain waveform feature recognition, Wavelet Packet Transform (WPT), and wavelet scalogram-based classification [3,4,5,6]. However, most existing methods still rely on a single feature form or a single input pathway, making it difficult to simultaneously capture statistical characteristics, spectral distributions, local time–frequency structures, and waveform-edge information. For fault scenarios with similar waveform characteristics, such as icing, wind deviation, and external damage, single-representation methods are more likely to suffer from insufficient discriminative information.

To enhance the deep representation capability of non-stationary transient signals, converting one-dimensional time series into two-dimensional images and combining them with Convolutional Neural Networks (CNNs) has become an effective approach in power fault diagnosis. Methods combining Fast Fourier Transform (FFT), Gramian Angular Field (GAF), and CNNs have been applied to fault identification of High-Voltage Direct Current (HVDC) transmission lines [7]. GAF constructs two-dimensional images through polar-coordinate mapping and angular relationships, thereby preserving the temporal correlation structure of the original sequence [8]. In addition, converting fault signals into two-dimensional scalograms using Continuous Wavelet Transform (CWT) and then feeding them into two-dimensional CNNs can also capture time–frequency features [9]. It should be noted that scalograms mainly emphasize time–frequency energy distribution, whereas GAF focuses more on angular-correlation structures among sample points. For transmission line fault traveling-wave signals, the Gramian Angular Summation Field (GASF) is more suitable for describing global correlation structures, while the Gramian Angular Difference Field (GADF) is more effective in characterizing local variations and difference structures. Therefore, using only a single image input or a single deep-feature classification path makes it difficult to fully exploit the complementary representations of GASF and GADF.

In addition to insufficient feature representation, historical transmission line fault samples also suffer from class imbalance. Owing to differences in fault occurrence probability, operating environments, and sample accumulation conditions, common faults such as lightning strikes usually have a relatively large number of samples, whereas minority-class faults such as bird-related incidents, icing, and external damage are relatively limited. If imbalanced samples are directly used for model training, the classifier tends to be biased toward majority classes, thereby weakening its ability to identify minority-class faults. The Synthetic Minority Over-sampling Technique (SMOTE) is a classical method for imbalanced classification problems [10]. The Borderline Synthetic Minority Over-sampling Technique (Borderline-SMOTE) further focuses on minority samples near classification boundaries and can enhance the representation capability of minority classes in boundary regions [11]. Related studies have also shown that combining SMOTE with deep neural networks can improve diagnostic performance under imbalanced sample conditions [12].

On the other hand, multi-domain explicit features can describe fault traveling-wave characteristics from different perspectives. However, high-dimensional candidate features may still contain redundant, weakly relevant, and highly correlated components. Directly feeding all features into a model may increase training complexity and degrade generalization performance. Optimized classification models have been used for transmission line fault classification and localization, indicating that the joint design of optimization strategies and classification models can improve fault diagnosis performance [13]. The Polar Lights Optimizer (PLO) is a recently proposed swarm intelligence optimization algorithm that has shown effectiveness in image segmentation and feature selection tasks, making it suitable for high-dimensional feature subset search [14]. Moreover, at the feature fusion level, the Multi-Head Attention mechanism can establish feature correlations from different subspaces and adaptively enhance key feature components [15], which makes it suitable for fusion modeling of deep image features and explicit features.

To address the above issues, this study proposes a transmission line fault type identification method that integrates Polar Lights Optimizer (PLO)-based feature selection with a Gramian Angular Field (GAF) attention fusion network. Historical fault traveling-wave data are first used to construct a labeled dataset, and the Borderline Synthetic Minority Over-sampling Technique (Borderline-SMOTE) is applied only to the training set to alleviate class imbalance. Multi-domain explicit features are then extracted from the time, frequency, time–frequency, and waveform-edge domains, and PLO is used to select key discriminative features. Meanwhile, the preprocessed traveling-wave sequences are encoded into dual-branch Gramian Angular Summation Field (GASF) and Gramian Angular Difference Field (GADF) images. A Gramian Angular Field–Parallel Convolutional Neural Network–Attention (GAF-PCNN-AT) model is further constructed to achieve adaptive fusion of deep image features and PLO-selected explicit features.

Existing GAF-based fault diagnosis studies have demonstrated that one-dimensional fault signals can be transformed into two-dimensional angular-correlation representations for image-based classification [7,8]. However, most of these methods still focus on GAF image construction, classifier-backbone enhancement, or single/directly concatenated image inputs, whereas optimized explicit physical features and attention-based heterogeneous fusion are not jointly considered. Compared with existing GAF-CNN-based fault diagnosis studies, this study further constructs a feature-optimized heterogeneous representation framework for imbalanced transmission line fault type identification.

The main contributions are summarized as follows. First, GASF and GADF are used to characterize global correlation structures and local difference patterns of traveling-wave signals, respectively, and are learned through two independent convolutional branches. Second, PLO-based feature selection is introduced to retain compact and discriminative explicit physical features from the 42-dimensional candidate feature set before heterogeneous feature fusion, thereby reducing redundant feature interference. Third, a three-branch GAF-PCNN-AT model is constructed to achieve attention-based adaptive fusion of deep GAF image features and selected explicit features. This design provides the practical insight that optimized multi-domain physical features can complement GAF-based deep image representations and improve fault type identification under imbalanced and morphologically similar transmission line fault samples.

## 2. Fault Data Preprocessing and Sample Balancing

### 2.1. Preprocessing of Transmission Line Fault Traveling-Wave Data

Distributed online monitoring devices installed on transmission lines are used to monitor the operating status of transmission lines in real time. These devices collect, store, and upload current traveling-wave signals generated during fault transients through monitoring terminals deployed along transmission lines, thereby providing the raw data basis for fault cause analysis and fault type identification. Based on the existing distributed online monitoring system, transient traveling-wave current waveforms and their corresponding fault labels can be obtained when transmission line faults occur, providing data support for subsequent fault sample construction, feature extraction, and model training.

**Figure 1:**
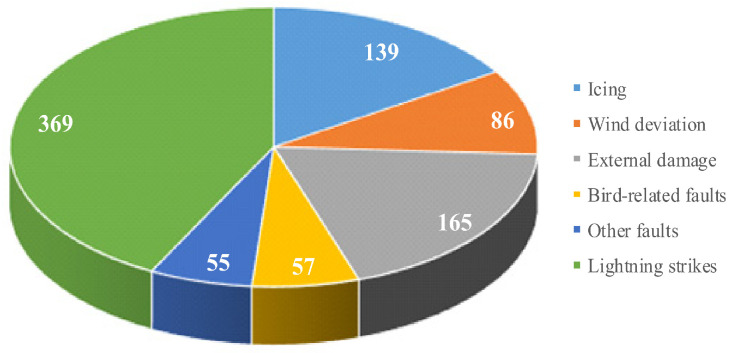
Statistics of six types of transmission line faults.

**Figure 2:**
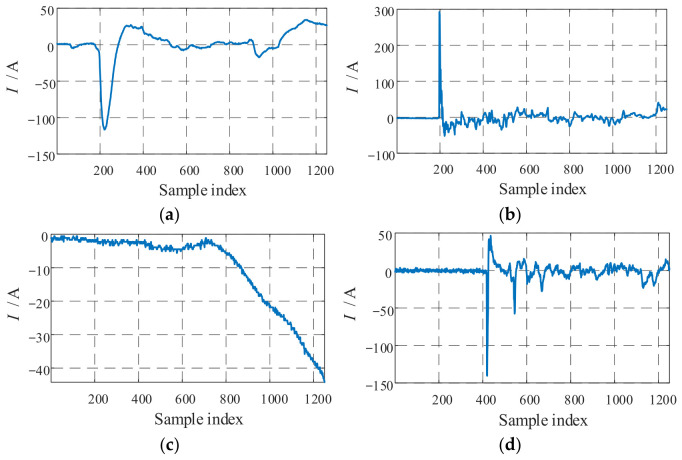
Typical traveling-wave waveforms of selected fault types: (**a**) typical traveling-wave sample of an icing fault; (**b**) typical traveling-wave sample of a wind deviation fault; (**c**) typical traveling-wave sample of an external damage fault; (**d**) typical traveling-wave sample of a bird-related fault.

Historical fault records of 500 kV and above transmission lines from 2018 to 2021 contain six types of faults, including icing, wind deviation, external damage, bird-related faults, other faults, and lightning strikes. In this study, 871 valid historical fault traveling-wave samples were collected, including 139 icing samples, 86 wind deviation samples, 165 external damage samples, 57 bird-related fault samples, 55 other fault samples, and 369 lightning strike samples. Among them, lightning strike samples account for the largest proportion, approximately 42.36%, whereas bird-related faults and other faults contain relatively fewer samples, with only 57 and 55 samples, respectively. This indicates an evident class imbalance in the fault dataset. The distribution of the six fault categories is shown in Figure 1. To meet the confidentiality requirements of field operation data, the actual transmission line names involved in this study are anonymized, while only the fault type, waveform morphology, and sample statistical information are retained. Typical traveling-wave waveforms of selected anonymized fault samples are shown in Figure 2.

To improve the accuracy of fault type identification, the transient traveling-wave current waveforms recorded by distributed online monitoring devices are preprocessed before feature extraction. First, the raw traveling-wave data are cleaned by removing abnormal values, erroneous data, and invalid records. Second, the retained samples are uniformly organized to form a labeled fault database, ensuring the quality and accuracy of the sample data. Finally, the six fault categories are processed in a consistent format, providing unified data inputs for subsequent sample balancing, image construction, and feature extraction.

### 2.2. Six-Class Fault Sample Balancing Based on Borderline-SMOTE

The original sample statistics show evident differences in the number of samples among different fault types. If imbalanced samples are directly used for model training, the classifier tends to be biased toward majority-class samples during parameter learning, thereby weakening its ability to identify minority-class faults. To reduce the adverse effect of class imbalance on fault identification performance, Borderline Synthetic Minority Over-sampling Technique (Borderline-SMOTE) is adopted in this study to balance the six fault categories in the training set during the model training stage.

Borderline-SMOTE is an improved version of the conventional Synthetic Minority Over-sampling Technique (SMOTE). Its core idea is to perform targeted over-sampling near the classification boundary of minority-class samples, thereby increasing the sample density of minority classes in boundary regions. Related studies have shown that introducing over-sampled datasets into power equipment fault diagnosis can alleviate the influence of insufficient minority-class samples on data-driven diagnostic models [16]. This method first categorizes minority-class samples into Safe, Danger, and Noise samples according to the proportion of majority-class samples among their nearest neighbors. Let *m* denote the number of nearest neighbors of a minority-class sample, and let *m*′ denote the number of majority-class samples among these neighbors. When *m*′ < *m*/2, the sample is classified as a Safe sample, indicating that minority-class samples still dominate its neighborhood, and it is usually not selected as a key over-sampling target. When *m*/2 ≤ *m*′ < *m*, the sample is classified as a Danger sample, indicating that it is located near the class boundary and is most susceptible to interference from majority-class samples; therefore, it is selected as the main over-sampling target in Borderline-SMOTE. When *m*′ = *m*, the sample is classified as a Noise sample, indicating that all its neighbors are majority-class samples; such samples are usually regarded as noisy samples and are not used for over-sampling.

**Figure 3:**
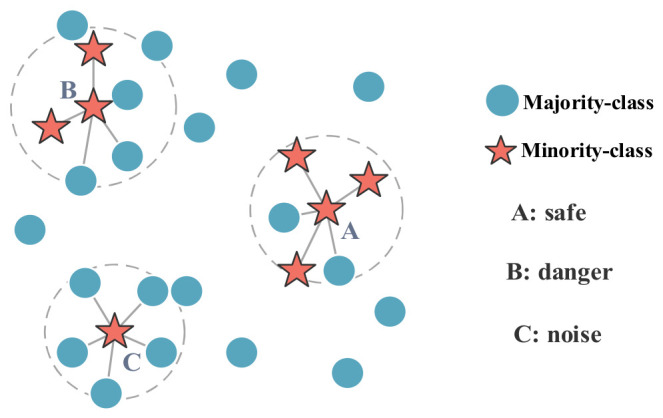
Schematic illustration of sample categorization in Borderline-SMOTE.

As shown in Figure 3, A, B, and C correspond to Safe, Danger, and Noise samples, respectively. For sample A, minority-class samples account for the majority of its neighborhood, so it is generally not used to generate new samples. For sample B, majority-class samples account for a relatively high proportion of its neighborhood but do not occupy the entire neighborhood, indicating that it is located near the class boundary and should be treated as a key over-sampling object in Borderline-SMOTE. For sample C, all nearest neighbors are majority-class samples, so it is usually regarded as a noisy sample and excluded from new sample generation. Unlike conventional SMOTE, which uniformly generates synthetic samples for all minority-class samples, Borderline-SMOTE mainly over-samples Danger samples. This strategy makes the generated samples more concentrated near class boundaries and improves the boundary representation capability of minority-class samples.

For minority-class samples classified as Danger samples, new synthetic samples are generated by linear interpolation. Let *x_i_* denote a Danger minority-class sample, and let *x_zi_* denote one of its minority-class nearest neighbors. A new sample can then be generated by linear interpolation as follows:

(1) $$x_{\mathrm{new}}=x_{i}+\lambda (x_{zi}-x_{i})$$

where λ∈(0,1) is a random coefficient.

Using the above procedure, new synthetic samples can be generated within the neighborhood of minority-class samples, thereby gradually supplementing the number of minority-class fault samples. During model training, Borderline-SMOTE is applied only to the six fault categories in the training set, so that the number of training samples in each class tends to be balanced. This provides a more balanced data basis for subsequent Gramian Angular Field image construction, explicit feature extraction, and fault classification model training.

## 3. Multi-Representation Construction and Feature Selection Method Based on GAF-PCNN-AT

### 3.1. Construction of Multi-Domain Explicit Features

In recent years, feature engineering studies for transmission line fault detection and diagnosis have shown that reasonably constructing and selecting features related to fault mechanisms can improve model interpretability, diagnostic accuracy, and training efficiency [17]. Fault traveling-wave signals are characterized by strong abruptness, short duration, and wide frequency-band distribution. Their class differences are reflected not only in amplitude statistics but also in spectral energy distribution, local time–frequency structures, and wavefront-edge morphology. To avoid insufficient characterization of transient differences by a single feature domain, this study takes multi-domain explicit features as structured physical priors and constructs a candidate feature set from four aspects: time domain statistics, frequency domain spectral distribution, Wavelet Packet Decomposition (WPD)-based time–frequency representation, and waveform-edge morphology. This provides an input basis for subsequent Polar Lights Optimizer (PLO)-based feature subset search and heterogeneous representation fusion.

A total of 42-dimensional explicit features are constructed in this study, including 17 time domain features, 4 frequency domain features, 18 time–frequency domain features, and 3 waveform morphology features. This feature system considers statistical characteristics, spectral distribution characteristics, local time–frequency characteristics, and waveform-edge morphology. It has good interpretability and provides a stable input basis for subsequent feature selection and multi-representation fusion.

#### 3.1.1. Time Domain Statistical Features

Time-domain features are mainly used to characterize the amplitude scale, fluctuation intensity, distribution dispersion, and pulse sharpness of fault traveling waves. In this study, the maximum value, minimum value, mean value, peak value, peak-to-peak value, average amplitude, root mean square, skewness, kurtosis, and several dimensionless ratio-type features are extracted from the original waveform to describe differences in amplitude response and pulse morphology among different fault samples.

These features directly reflect the overall amplitude level, fluctuation intensity, and impact characteristics of fault waveforms. Furthermore, skewness, kurtosis, and ratio-type indicators can reveal wavefront abruptness and waveform peak characteristics. For transmission line fault traveling waves, time domain statistical features are among the most fundamental and interpretable representations, providing stable statistical inputs for subsequent classification models.

#### 3.1.2. Frequency Domain Spectral Distribution Features

To further characterize differences in the spectral structure of fault samples, frequency domain analysis is performed on the traveling-wave signals, and frequency domain features such as mean frequency, centroid frequency, root mean square frequency, and total harmonic distortion are extracted. Compared with time domain features, frequency domain features can describe differences among faults in high-frequency disturbances, dominant frequency-band positions, and spectral dispersion from the perspective of spectral energy distribution.

For fault traveling waves, frequency domain features are particularly suitable for distinguishing wavefront abruptness, local high-frequency oscillation levels, and spectral energy concentration intervals. Therefore, they can complement time domain statistical features and improve the multi-dimensional discriminative capability of fault samples.

#### 3.1.3. Time–Frequency Domain Features Based on Wavelet Packet Decomposition

To characterize the non-stationary distribution of fault traveling-wave signals in the time–frequency domain, Wavelet Packet Decomposition (WPD) is applied to perform a three-level multi-scale decomposition of the original traveling-wave current. Unlike conventional wavelet transform, which further decomposes only the low-frequency component, WPD recursively decomposes both low-frequency and high-frequency components. Therefore, it is more suitable for characterizing complex features in fault traveling waves, such as multi-band coupling, spectral abrupt changes, and local energy redistribution. In transmission line fault analysis, previous studies have used Wavelet Packet Transform (WPT) to extract fault transient information and waveform entropy features, demonstrating its suitability for describing non-stationary transmission line fault signals [5].

**Figure 4:**
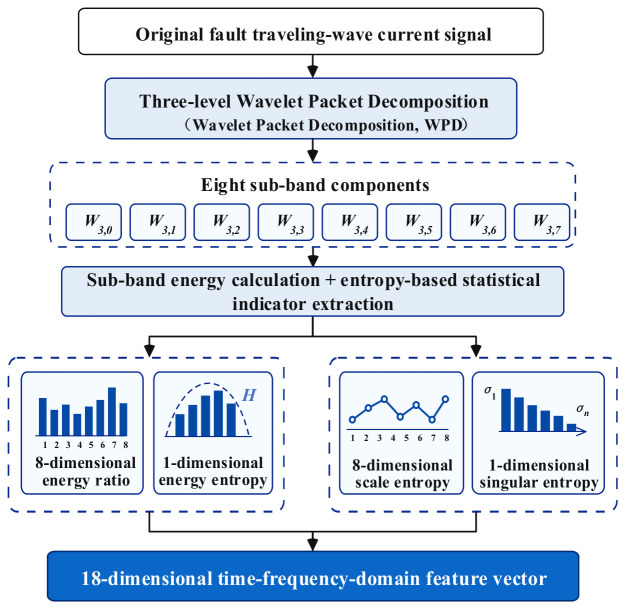
Schematic diagram of time–frequency feature construction based on Wavelet Packet Decomposition (WPD).

After the three-level sub-band components are obtained, time–frequency domain statistical indicators, including energy ratios, energy entropy, scale entropy, and singular entropy, are further constructed from each sub-band to form an 18-dimensional time–frequency domain feature vector for fault identification. To highlight the progressive process of “signal decomposition–sub-band representation–entropy-based statistics”, the overall feature construction process, including three-level WPD, sub-band energy calculation, and entropy-based indicator extraction, is summarized in Figure 4.

After three-level WPD is performed on a fault traveling-wave sequence, eight sub-bands can be obtained. Let *D*_3_,*_i_*(*j*) denote the *j*-th wavelet packet coefficient of the *i*-th sub-band at the third decomposition level. The energy of this sub-band and its normalized energy ratio can be expressed as

(2) $$E_{3,i}=\sum_{j=1}^{N_{i}}{\left|D_{3,i}(j)\right|}^{2},\;\rho_{i}=\frac{E_{3,i}}{\sum_{k=0}^{7}E_{3,k}},\;i=0,1,\cdots ,7$$

where *D*_3_,*_i_*(*j*) denotes the *j*-th coefficient of the *i*-th wavelet packet sub-band at the third level, *N_i_* denotes the number of wavelet packet coefficients in this sub-band, *E*_3,*i*_ denotes the energy of the *i*-th sub-band at the third decomposition level, *k* denotes the sub-band index used in the total-energy summation, and *ρ_i_* denotes the normalized energy ratio of the *i*-th sub-band. Accordingly, eight-dimensional wavelet packet energy-ratio features can be obtained. Furthermore, this study adopts the information entropy form to describe the dispersion degree of energy or singular-value distributions, and its unified expression is given by

(3) $$H(u)=-\sum_{l=1}^{L}{\bar{u}}_{l}\mathrm{lg}{\bar{u}}_{l},\;{\bar{u}}_{l}=\frac{u_{l}}{\sum_{r=1}^{L}u_{r}}$$

where ***u*** denotes a non-negative feature vector used for entropy calculation, *L* denotes the dimension of ***u***, *u_ℓ_* denotes the *ℓ*-th component of ***u***, and ${\bar{u}}_{l}$ denotes the *ℓ*-th normalized component. On this basis, wavelet energy entropy, wavelet scale entropy, and wavelet singular entropy can be constructed by using wavelet packet sub-band energy, scale-spectrum energy, and singular-value distribution as inputs, respectively. The above energy-ratio and entropy-based indicators jointly constitute the 18-dimensional time–frequency domain features used in this study.

#### 3.1.4. Waveform-Edge Features

In addition to statistical and time–frequency features, fault traveling waves also exhibit strong discriminative characteristics in terms of pulse duration, edge variation rate, and wavefront width. To further enhance the characterization of waveform geometry, this study extracts three waveform-edge features, namely pulse width, fall time, and rise time.

These features can more directly reflect the morphological evolution of the fault wavefront and wave tail, and they provide important complementary information for distinguishing fault samples with similar statistical distributions but different edge evolution patterns. Accordingly, a 42-dimensional explicit feature vector is finally obtained, providing the input basis for subsequent Polar Lights Optimizer (PLO)-based feature selection and the explicit feature mapping branch.

### 3.2. Traveling-Wave Sequence Image Encoding Based on Gramian Angular Field

To convert one-dimensional fault traveling-wave sequences into two-dimensional image representations suitable for convolutional network learning, this study adopts the Gramian Angular Field (GAF) for time-series image encoding. This part focuses on constructing dual-image inputs based on the Gramian Angular Summation Field (GASF) and the Gramian Angular Difference Field (GADF), providing image-based samples for the parallel convolutional feature extraction branch in Section 4. GAF was first proposed by Wang and Oates. Its basic idea is to map a normalized time series into the polar coordinate space and then construct a Gramian matrix through angular summation and difference relationships, thereby preserving the temporal dependence and correlation structure of the original sequence in a two-dimensional image [8]. In recent years, this method has been applied to power fault diagnosis and equipment diagnosis scenarios and has shown good image representation capability [18].

Let the normalized sequence be denoted as ${\tilde{x}}_{i}\in [-1,1]$. Its polar coordinate mapping can be expressed as

(4) $$\phi_{i}=\arccos({\tilde{x}}_{i}),\;\;\;r_{i}=\frac{i}{N}$$

where ${\tilde{x}}_{i}$ denotes the *i*-th normalized sampling point of the traveling-wave sequence, $\phi_{i}$ denotes the corresponding angular coordinate in the polar-coordinate representation, *r_i_* denotes the normalized radial coordinate, and *N* denotes the sequence length.

On this basis, the GASF and GADF can be constructed as follows:

(5) $$\left\{\begin{array}{l} {\mathrm{GASF}}_{i,j}=\cos(\phi_{i}+\phi_{j})\\ {\mathrm{GADF}}_{i,j}=\sin(\phi_{i}-\phi_{j}) \end{array}\right.$$

where GASF*_i_*_,*j*_ and GADF*_i_*_,*j*_ denote the elements in the *i*-th row and *j*-th column of the GASF and GADF matrices, respectively, and $\phi_{i}$ and $\phi_{j}$ denote the angular coordinates corresponding to the *i*-th and *j*-th sampling points.

Among them, GASF mainly reflects the global correlation structures among sample points and highlights the overall evolution trend of the fault waveform. In contrast, GADF is more sensitive to difference relationships and local variation patterns among sample points, thereby enhancing edge abrupt changes, local oscillations, and fine-grained disturbance information. Constructing GASF and GADF images simultaneously for the same fault sample can enhance the spatial representation capability of traveling-wave signals from complementary perspectives.

In this study, the one-dimensional traveling-wave sequence used for GAF encoding contains 1250 sampling points. Before GAF encoding, the one-dimensional traveling-wave sequence is normalized and mapped into the polar-coordinate space. The resulting GASF and GADF matrices are normalized, converted into pseudo-color RGB images using the jet colormap, and then resized to 227 × 227, resulting in 227 × 227 × 3 inputs for the CNN branches. The image size of 227 × 227 is selected as a compromise between representation completeness and computational cost. A smaller image size may reduce local transient details of the fault wavefront, whereas an excessively large image size increases memory consumption and training complexity without necessarily improving the separability of fault categories. Therefore, 227 × 227 is adopted to preserve the main angular-correlation structure of the traveling-wave sequence while maintaining reasonable computational efficiency.

Based on the above encoding strategy, each fault sample is mapped into a pair of GASF/GADF dual-branch images, which provide the input basis for subsequent parallel convolutional branches to extract deep spatial features. Specifically, GASF focuses on representing the global correlation structure and overall evolution trend of the traveling-wave sequence in the temporal dimension, whereas GADF emphasizes local difference patterns, wavefront abrupt changes, and fine-grained disturbances between adjacent sampling points. Compared with a single image representation, dual-branch GAF encoding can characterize fault traveling-wave features from two complementary perspectives, namely global correlation and local difference, thereby enhancing the separability among similar fault categories.

### 3.3. Explicit Feature Subset Search Based on PLO

Although the 42-dimensional explicit features characterize fault traveling-wave signals from multiple perspectives, redundant features, weakly contributing features, and highly correlated features are still inevitable. If all features are directly fed into the classification model, the training complexity may increase, and redundant noise may be introduced, thereby weakening the generalization performance of the model. Therefore, the Polar Lights Optimizer (PLO) is introduced in this study to select explicit features and identify the feature subset that contributes most to fault classification from the 42 candidate features.

PLO was proposed by Yuan et al. in 2024 [14] as a swarm intelligence optimization algorithm based on the motion mechanism of polar lights. The original study presented a binary modeling strategy for feature selection and verified its effectiveness in benchmark tests and feature selection tasks. Compared with traditional exhaustive search or simple filtering methods, PLO has three advantages in feature selection. First, it can balance global exploration and local exploitation in a relatively large search space. Second, it can control the size of the feature subset while maintaining classification performance. Third, it can effectively reduce redundant interference in explicit features and improve the utilization efficiency of key features in the subsequent fusion model [14].

Let the feature selection vector be defined as

(6) $$s=[s_{1},s_{2},\cdots ,s_{42}],\;s_{i}\in \{0,1\}$$

where *s_i_* = 1 indicates that the *i*-th feature is selected, and *s_i_* = 0 indicates that the *i*-th feature is discarded. To jointly consider classification performance and feature dimensionality, the fitness function is constructed as

(7) $$J(s)=\alpha (1-{\mathrm{Acc}}_{\mathrm{val}}(s))+\beta \frac{|s{|}_{0}}{42}$$

where Accval(***s***) denotes the classification accuracy of feature subset s evaluated on training-side validation data, |***s***|_0_ denotes the number of selected features, and *α* and *β* are the weighting coefficients of the classification-error term and the feature-size penalty term, respectively. In this study, *α* is set to 1 and *β* is set to 0.08, so that training-side validation accuracy is treated as the primary optimization objective while the feature-size penalty is used to reduce redundant selected features.

Within this framework, PLO iteratively searches the binary feature subsets and gradually approaches the optimal solution by minimizing the fitness value. After PLO-based selection, 18 optimized features are finally selected from the original 42-dimensional explicit feature set and used as the input to the subsequent explicit feature mapping branch. This process reduces feature redundancy and improves the complementarity between explicit features and image representations.

### 3.4. Multi-Representation Input Construction for GAF-PCNN-AT

After explicit feature construction, GAF image encoding, and PLO-based feature selection are completed, a multi-representation input form is further established for six-class fault identification, providing the input basis for feature fusion and classification in the subsequent GAF-PCNN-AT model. Related studies have shown that attention-based feature fusion can adaptively enhance key feature responses and improve diagnostic performance in complex fault scenarios [19].

For each fault sample, three input branches are constructed in this study:

(1) A GASF image representing the global correlation structure;
(2) A GADF image representing the local difference pattern;
(3) An 18-dimensional PLO-selected explicit feature vector representing physical statistical characteristics.

Specifically, the GASF branch is used to capture the overall correlation and trend variation in fault traveling waves in the temporal dimension, whereas the GADF branch is used to enhance wavefront abrupt changes, local oscillations, and fine-grained disturbance information. The PLO-selected multi-domain explicit features retain physically meaningful information related to time domain statistics, spectral distribution, time–frequency structures, and waveform edges. Through this three-input design involving “global correlation structure–local difference pattern–multi-domain explicit features”, the model can avoid insufficient fault information characterization caused by a single representation and achieve collaborative modeling and joint discrimination of the six fault categories.

In summary, Section 3 completes the construction process from raw fault traveling-wave data to multi-representation fusion inputs. A heterogeneous feature input set consisting of GASF global images, GADF local difference images, and PLO-selected multi-domain explicit features is formed. This input set not only retains the deep image structure of traveling-wave signals but also embeds physically interpretable statistical, spectral, time–frequency, and waveform-edge information, providing the basis for adaptive fusion-based identification in the GAF-PCNN-AT model presented in Section 4.

## 4. Fault Identification Model Based on GAF-PCNN-AT

### 4.1. Overall Model Architecture

Based on the preceding fault traveling-wave sample balancing, multi-domain explicit feature construction, GASF/GADF dual-image encoding, and PLO-based feature selection, this study further constructs a GAF-PCNN-AT model for six-class fault identification. In GAF-PCNN-AT, GAF denotes Gramian Angular Field image encoding, PCNN denotes Parallel Convolutional Neural Network, and AT denotes the attention mechanism. The model takes GASF images, GADF images, and PLO-selected explicit features as multi-representation inputs. Through collaborative modeling of the dual-branch image module and the explicit feature mapping branch, automatic identification of six fault categories, namely icing, wind deviation, external damage, bird-related faults, other faults, and lightning strikes, is achieved.

From the perspective of the overall architecture, the proposed model mainly consists of a dual-branch Gramian Angular Field image representation module, an explicit feature branch, a feature fusion module, a multi-head attention enhancement module, and a classification output module. Among them, GASF and GADF encode fault traveling-wave samples from the perspectives of global correlation structure and local difference pattern, respectively, and are suitable for extracting deep spatial features through parallel convolutional branches. The PLO-selected multi-domain explicit features retain physically interpretable information related to time domain statistics, spectral distribution, time–frequency structures, and waveform edges, and can serve as an important supplement to image representations. Subsequently, the three high-level features are fused at the feature level, and a multi-head attention mechanism is introduced to adaptively enhance key feature components, thereby improving the discriminative capability of the model for different fault categories.

Let the GASF image input, GADF image input, and PLO-selected explicit features be denoted as ***I***_GASF_, ***I***_GADF_, and f*, respectively. The overall mapping relationship of the proposed model can be expressed as

(8) $$\hat{y}=F\left(I_{\mathrm{GASF}},I_{\mathrm{GADF}},f^{*}\right)$$

where F(⋅) denotes the mapping function of the constructed GAF-PCNN-AT classification model, and $\hat{y}$ denotes the predicted fault category.

**Figure 5:**
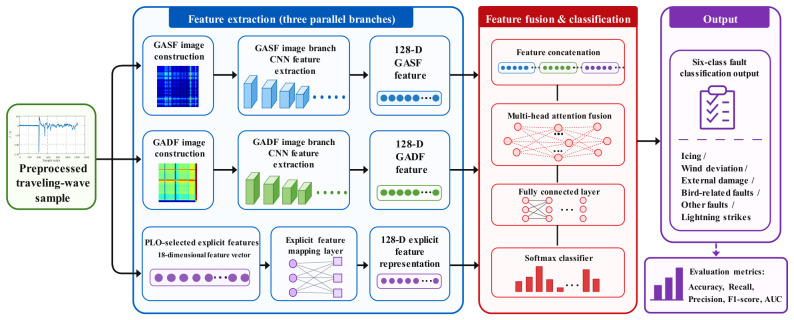
Overall architecture and fault identification workflow of the proposed GAF-PCNN-AT model.

Based on the above architecture, the overall architecture and fault identification workflow of the proposed GAF-PCNN-AT model are shown in Figure 5. The model consists of three parallel feature extraction branches, namely the GASF image branch, the GADF image branch, and the PLO-selected explicit feature branch. The GASF and GADF branches extract 128-dimensional high-level image features from global correlation structures and local difference patterns, respectively, while the explicit feature branch maps the 18-dimensional PLO-selected explicit feature vector into a 128-dimensional explicit feature representation. The three high-level representations are then concatenated at the feature level and adaptively enhanced by the multi-head attention module. Finally, the fused features are passed through a fully connected layer and a Softmax classifier to obtain the six-class fault classification output.

Compared with a single-GAF-image input model, the proposed model simultaneously characterizes the global correlation structure and local difference pattern of traveling-wave sequences through the GASF/GADF dual-branch structure. Compared with methods relying only on manually extracted explicit features, the proposed model can further exploit deep spatial correlation features in fault traveling waves. Compared with simple feature concatenation, the introduction of PLO-based feature selection and the multi-head attention mechanism enables the model to reduce redundant inputs while adaptively enhancing key discriminative features. Therefore, the constructed GAF-PCNN-AT model forms a complete identification loop from fault traveling-wave input, multi-representation feature extraction and high-level mapping, attention-based fusion, to fault category output.

### 4.2. GASF/GADF Parallel Convolutional Feature Extraction Branches

Based on the GASF/GADF dual-image inputs constructed in Section 3, parallel convolutional feature extraction branches are further designed to separately learn deep spatial textures and structural patterns from the two types of GAF images. The two branches take GASF and GADF images as inputs, respectively. They share the same network topology, whereas their parameters are updated independently, enabling the model to extract the global correlation structure represented by GASF and the local difference pattern represented by GADF in different feature subspaces. Therefore, the dual-branch parallel convolutional structure can preserve the complementarity of the two image representations during feature extraction and provide richer high-level image features for subsequent feature fusion and attention enhancement.

Each image branch consists of an image input layer, a convolutional layer, a batch normalization layer, a nonlinear activation layer, a pooling layer, and a fully connected mapping layer. The input image size is uniformly set to 227 × 227 × 3. First, shallow spatial features are extracted using a convolutional layer with a kernel size of 7 × 7, 64 kernels, and a stride of 2. Then, a batch normalization layer and a rectified linear unit (ReLU) activation layer are used to improve training stability and nonlinear representation capability. A max-pooling layer is subsequently adopted for local dimensionality reduction and key texture response preservation. Finally, a fully connected mapping layer is used to obtain a 128-dimensional high-level image feature representation.

Let the high-level features extracted by the GASF and GADF branches be denoted as ***h****_s_* and ***h****_d_*, respectively. Then,

(9) $$h_{s}=\Phi_{s}\left(I_{\mathrm{GASF}}\right),\;h_{d}=\Phi_{d}\left(I_{\mathrm{GADF}}\right)$$

where $\Phi_{s}(\cdot )$ and $\Phi_{d}(\cdot )$ denote the nonlinear mapping functions of the GASF and GADF parallel convolutional branches, respectively, and ***h****_s_* and ***h****_d_* denote the 128-dimensional high-level image features output by the two image branches.

**Figure 6:**
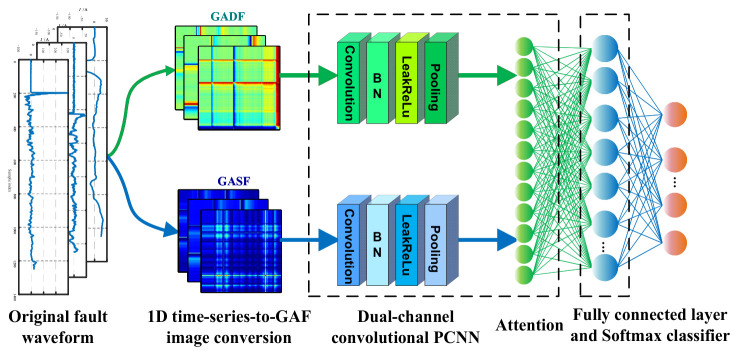
Schematic diagram of the dual-branch GASF/GADF image representation and classification structure.

This study adopts a dual-branch parallel convolutional structure rather than directly concatenating GASF and GADF into a single input path. The rationale is as follows. First, GASF and GADF correspond to the global correlation structure and the local difference pattern, respectively, and therefore belong to different feature representation subspaces. Second, the two branches share the same network topology but update their convolutional parameters independently. This design avoids mutual interference between heterogeneous images during shallow feature extraction and enables the network to learn more targeted fault pattern representations from each GAF representation. Third, no explicit cross-attention is introduced between the GASF and GADF image branches at the input or shallow-convolution stage. Instead, cross-representation interaction is performed after high-level feature mapping: the GASF feature, GADF feature, and PLO-selected explicit feature are concatenated at the feature level and then adaptively enhanced by the multi-head attention module. Thus, the dual-branch structure exploits the complementarity of GASF and GADF through independent branch learning, feature-level fusion, and attention-based adaptive weighting. The corresponding dual-branch GASF/GADF image representation and classification structure is illustrated in Figure 6.

### 4.3. PLO-Selected Multi-Domain Explicit Feature Mapping Branch

Based on the 18-dimensional multi-domain explicit features selected by PLO in Section 3, an explicit feature mapping branch is constructed to project the low-dimensional physical-feature vector into a high-level representation space consistent with the image branches. This branch is used to retain physically interpretable information related to time domain statistics, spectral distribution, time–frequency structures, and waveform edges of fault traveling waves, and provides complementary information for subsequent multi-representation feature fusion.

Compared with directly using the full 42-dimensional feature set as input, the 18-dimensional multi-domain explicit features selected by PLO can reduce the interference caused by redundant and weakly relevant features during model training, enabling the explicit feature branch to focus more on key information closely related to fault class discrimination. Specifically, this branch consists of a feature input layer, two fully connected layers, and corresponding ReLU activation layers. It maps the 18-dimensional selected explicit features into a 128-dimensional high-level feature representation, thereby forming three high-level representation inputs with consistent dimensionality and similar semantic levels together with the outputs of the GASF/GADF image branches. Let the PLO-selected explicit feature vector be denoted as $f^{*}\in {\mathbb{R}}^{18}$. The output of the explicit feature mapping branch can be expressed as

(10) $$h_{f}=\Psi \left(f^{*}\right)$$

where Ψ(⋅) denotes the nonlinear mapping function of the explicit feature mapping branch, and ***h****_f_* denotes the 128-dimensional feature representation obtained after high-level mapping of the PLO-selected multi-domain explicit features. After this process, the selected explicit features are projected into a representation space at the same level as the image branches, thereby providing the basis for subsequent feature-level fusion.

### 4.4. Feature Fusion and Multi-Head Attention Enhancement Mechanism

After obtaining the GASF branch feature ***h****_s_*, the GADF branch feature ***h****_d_*, and the output of the explicit feature branch ***h****_f_*, the three high-level representations are uniformly fused at the feature level. Compared with data-level fusion, feature-level fusion can retain the deep representation capability of each branch while enabling more sufficient interactions among different types of information. Compared with decision-level fusion, feature-level fusion can more effectively establish correlations among multi-representation features before classification, making it more suitable for the collaborative modeling of image representations and multi-domain explicit features in this study.

In this study, concatenation is first adopted to fuse the three feature branches, which is expressed as

(11) $$z=\mathrm{Concat}\left(h_{s},h_{d},h_{f}\right)$$

where Concat(⋅) denotes the feature vector concatenation operation, and ***z*** denotes the concatenated multi-representation feature vector. After fusion, a fully connected mapping layer is used to compress the high-dimensional concatenated vector into a unified 128-dimensional common feature space, thereby reducing the influence caused by dimensional imbalance among different branch features.

However, the fused features obtained by simple concatenation may still contain redundant components and weakly contributing features. To further highlight the key information most relevant to fault classification, a multi-head attention mechanism is introduced after the fusion layer to adaptively weight the fused high-dimensional representation. Let the query, key, and value matrices obtained through linear transformations of the fused features be denoted as ***Q***, ***K***, and ***V***, respectively. The single-head attention calculation is expressed as

(12) $$\mathrm{Attention}(Q,K,V)=\mathrm{Softmax}\left(\frac{QK^{T}}{\sqrt{d_{k}}}\right)V$$

where *d_k_* denotes the dimension of the key vector. Multi-head attention jointly models feature dependencies in different subspaces through multiple parallel attention heads, and its output is expressed as

(13) $$\left\{\begin{array}{l} {\mathrm{head}}_{h}=\mathrm{Attention}(QW_{h}^{Q},KW_{h}^{K},VW_{h}^{V}),\;h=1,2,\cdots ,H\\ \mathrm{MultiHead}(Q,K,V)=\mathrm{Concat}({\mathrm{head}}_{1},{\mathrm{head}}_{2},\cdots ,{\mathrm{head}}_{H})W^{O} \end{array}\right.$$

where head*_h_* denotes the output of the *h*-th attention head, *H* denotes the number of attention heads; $W_{h}^{Q}$, $W_{h}^{K}$, and $W_{h}^{V}$ denote the query, key, and value mapping matrices corresponding to the *h*-th attention head, respectively; and $W^{O}$ denotes the output mapping matrix. In this study, a four-head attention structure is used to enhance the 128-dimensional fused features.

By introducing the multi-head attention mechanism, the model no longer assigns equal weights to all fused features. Instead, it can dynamically enhance more discriminative image patterns and explicit feature components according to the requirements of the classification task, while suppressing the interference of redundant information and weakly relevant components. For the six fault categories investigated in this study, this mechanism helps improve the identification capability for samples near confusing class boundaries, thereby enhancing the overall classification performance of the model on the current dataset.

### 4.5. Classification Output and Model Training

After feature fusion and attention enhancement, the model enters the classification output stage. Let the attention-enhanced high-level feature representation be denoted as ***a***. The output of the classification layer can be expressed as

(14) $$p=\mathrm{Softmax}\left(W_{c}a+b_{c}\right)$$

where ***W****_c_* and ***b****_c_* denote the weight matrix and bias vector of the classification layer, respectively, and ***p*** denotes the predicted probability vector corresponding to each fault category. For the six-class fault identification task considered in this study, the number of output layer nodes is set to six, corresponding to icing, wind deviation, external damage, bird-related faults, other faults, and lightning strikes.

To achieve supervised training, the cross-entropy loss function is adopted as the optimization objective. Let *N* denote the total number of samples, *C* denote the number of classes, *y_n_*_,*c*_ denote the true one-hot label, and *p_n_*_,*c*_ denote the predicted probability that the *n*-th sample belongs to the *c*-th class. The cross-entropy loss function is defined as

(15) $$L=-\frac{1}{N}\sum_{n=1}^{N}\sum_{c=1}^{C}y_{n,c}\log p_{n,c}$$

where L denotes the cross-entropy training loss. During training, the model gradually learns the nonlinear mapping relationships among GASF images, GADF images, explicit features, and fault categories by minimizing the above loss function.

In summary, the constructed GAF-PCNN-AT model implements a complete modeling process that includes dual-branch Gramian Angular Field image representation, explicit feature selection, and multi-head-attention-enhanced classification. On the basis of collaborative representation of multi-source feature information, the model can achieve high-accuracy identification of six transmission line fault categories.

## 5. Case Study

### 5.1. Fault Data Processing

This study conducts a case study based on transmission line fault traveling-wave samples. Six fault categories, namely icing, wind deviation, external damage, bird-related faults, other faults, and lightning strikes, are selected as the research objects, and their label codes are set as 1–6, respectively. A total of 871 valid historical fault traveling-wave samples are collected, including 139 icing samples, 86 wind deviation samples, 165 external damage samples, 57 bird-related fault samples, 55 other fault samples, and 369 lightning strike samples. To avoid information leakage from the sample balancing process into the test set, the original fault samples are first stratified into a real training set and a real test set at an approximate ratio of 8:2. Subsequently, Borderline-SMOTE is applied only to the training set to expand the samples, so that the number of training samples in each fault category is balanced to 295. The test set does not participate in any sample synthesis process and retains its real sample attributes. The numbers of test samples for icing, wind deviation, external damage, bird-related faults, other faults, and lightning strikes are 28, 17, 33, 11, 11, and 74, respectively, giving a total of 174 test samples.

**Figure 7:**
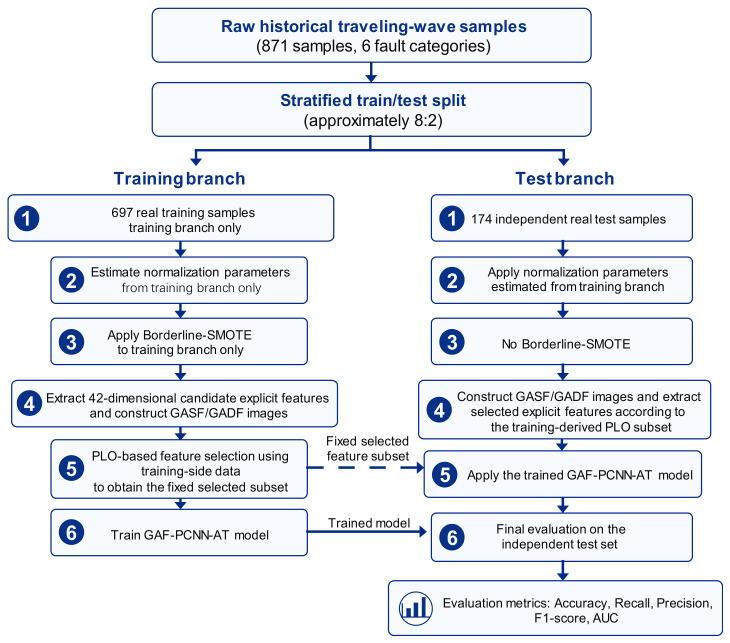
Data-processing and evaluation pipeline used to avoid information leakage. Numbers 1–6 indicate the sequential processing steps in the training and test branches.

To further clarify the experimental procedure and eliminate potential information leakage, the complete data-processing and evaluation pipeline is shown in Figure 7. After the stratified train/test split, the original fault samples are divided into a real training set and an independent real test set at an approximate ratio of 8:2. Normalization-parameter estimation, Borderline-SMOTE-based sample balancing, PLO-based feature selection, and model training are performed only within the training branch. The independent real test set does not participate in sample synthesis, normalization-parameter estimation, feature selection, hyperparameter tuning, or model training. After the selected feature subset and model settings are determined using the training-side data, the independent real test set is used only for final performance evaluation.

To comprehensively evaluate the fault identification performance of the model, Accuracy, average Recall, average Precision, average F1-score, and average area under the curve (AUC) are selected as evaluation indicators. Comparative analyses are conducted under unified experimental conditions for different fault identification scenarios, different feature selection methods, and different learning models.

### 5.2. Fault Feature Extraction and Diagnosis

#### 5.2.1. Fault Feature Extraction and PLO Selection Results

The fault features extracted in this study include four categories: time domain features, frequency domain features, time–frequency domain features, and waveform features. Specifically, the feature extraction results consist of 17 time domain features, 4 frequency domain features, 18 time–frequency domain features, and 3 waveform features, forming a 42-dimensional explicit feature set. By applying unified feature extraction to different fault traveling-wave samples, an explicit feature dataset for fault identification is finally constructed.

On this basis, the Polar Lights Optimizer (PLO) is used to select features from the 42 candidate features, aiming to reduce input redundancy and enhance the correlation between feature combinations and fault categories. The selection results show that 18 key features are finally retained, which are distributed across the time domain, frequency domain, time–frequency domain, and waveform feature spaces. The 18-dimensional explicit feature subset selected by PLO from the original 42-dimensional candidate feature set is shown in Table 1. For the selected wavelet packet-related features, the corresponding terminal sub-bands are further specified after the table to improve reproducibility.

For the wavelet packet-related features in Table 1, a three-level wavelet packet decomposition with db6 is used for the traveling-wave signal, producing eight terminal sub-bands. The k-th terminal sub-band corresponds to the MATLAB wavelet packet node [3, k−1]. The selected wavelet packet energy ratios are extracted from the 5th and 7th terminal sub-bands, corresponding to nodes [3, 4] and [3, 6], respectively. The selected wavelet packet scale entropies are extracted from the 2nd and 4th terminal sub-bands, corresponding to nodes [3, 1] and [3, 3], respectively. The wavelet singular entropy is calculated based on all eight terminal sub-bands rather than a single sub-band.

**Figure 8:**
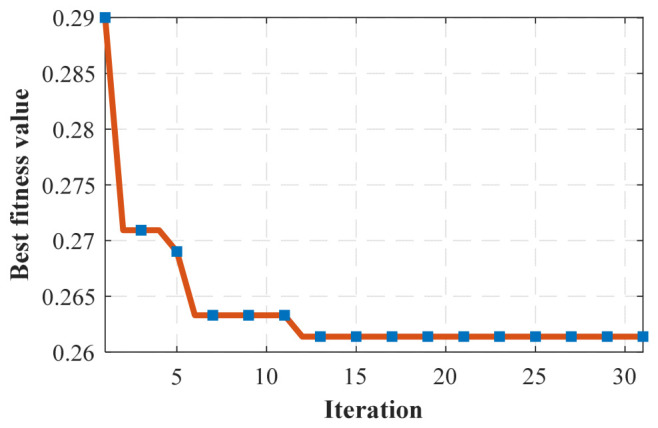
Convergence curve of the PLO-based feature selection process. The line and markers represent the best fitness value over iterations.

To further clarify the parameter settings and convergence behavior of the PLO-based feature selection process, the main PLO parameters are summarized in Table 2, and the corresponding convergence curve is shown in Figure 8.

As shown in Figure 8, the best fitness value decreases rapidly in the early iterations and then gradually reaches a stable plateau after approximately 12 iterations. This indicates that PLO can effectively search for a compact feature subset within the given iteration range. Since PLO-based feature selection is performed offline during model construction, its computational cost does not directly affect the online fault type identification stage.

The proposed PLO-based feature-optimized GAF-PCNN-AT model was simulated and validated using the MATLAB R2024a software platform. To improve the reproducibility of the model training process, the key model structure parameters and training hyperparameters are listed in Table 3.

The computational time was also recorded to evaluate the online applicability of the proposed method. The offline training time of the proposed GAF-PCNN-AT model was 977.70 s, approximately 16.29 min. During online fault type identification, the preprocessing stage, including 18-dimensional feature extraction, feature normalization, GASF construction, and GADF construction, required 108.66 ms per sample. The forward inference time of the trained network was 2.27 ms per sample. Therefore, the estimated end-to-end online time was 110.93 ms per sample. Since PLO-based feature selection and model training are completed offline, the online stage only requires feature extraction, GAF image construction, and forward inference. This response time can provide rapid fault type information for online monitoring, dispatching assistance, post-fault inspection guidance, and subsequent control decisions such as adaptive reclosing strategy support. These results indicate that the proposed method is computationally feasible for online transmission line fault type identification.

#### 5.2.2. Fault Diagnosis Results

**Figure 9:**
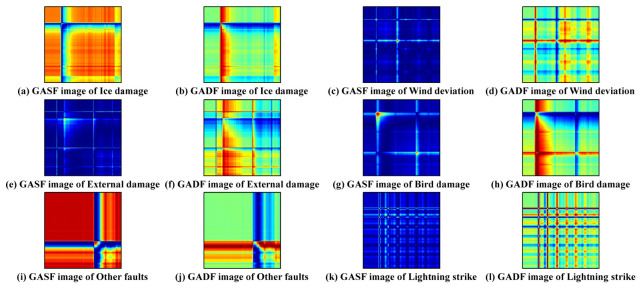
GASF/GADF image representations of representative test samples from six fault categories. The colors represent normalized GAF matrix values after pseudo-color mapping and do not indicate additional fault labels.

Under the above parameter settings, representative samples from the six fault categories in the test set were first selected to further demonstrate the multi-representation input characteristics of the proposed method. Their corresponding Gramian Angular Summation Field (GASF) and Gramian Angular Difference Field (GADF) image representations are shown in Figure 9.

According to the encoding mechanism of GAF, GASF focuses more on characterizing the global correlation structure and overall evolution trend among time-series samples through angular summation mapping, whereas GADF is more sensitive to local differences, edge abrupt changes, and fine-grained disturbances through angular difference mapping. The representative images in Figure 9 show certain differences in texture distribution, brightness concentration regions, and local structural patterns, indicating that the two image representations provide complementary descriptions of the same fault sample. Therefore, jointly using GASF and GADF as inputs can improve the model’s ability to identify complex fault categories.

**Figure 10:**
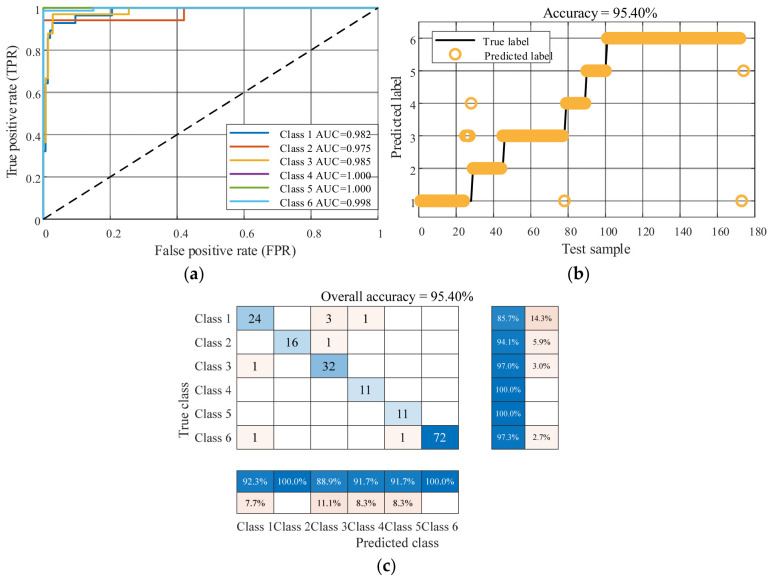
Diagnostic results of six fault types: (**a**) ROC curves; (**b**) prediction accuracy; (**c**) confusion matrix of fault identification. Different colors/markers distinguish fault classes, true/predicted labels, or confusion-matrix values, as indicated in each subfigure.

Furthermore, the prediction results, confusion matrix, and multi-class receiver operating characteristic (ROC) curves on the test set were used to comprehensively evaluate the identification performance of the proposed model, as shown in Figure 10. The corresponding evaluation indicators are listed in Table 4.

The results show that, among 174 real test samples, the proposed model correctly identified 166 samples, achieving an overall accuracy of 95.40%. Considering the representative GASF/GADF images in Figure 9 and their encoding mechanism, the dual-branch GAF representation can supplement fault traveling-wave information from the perspectives of global correlation structure and local difference pattern. In combination with the confusion matrix and ROC curves in Figure 10, these results demonstrate that the proposed method has strong overall identification capability for the six fault categories.

From the category-wise results, lightning strikes and wind deviation faults achieved relatively good identification performance, with Precision values of 100.00% and F1-scores of 98.63% and 96.97%, respectively. Bird-related faults and other faults both achieved a Recall of 100.00%, while their Precision values were 91.67%. In contrast, the Recall of icing faults was relatively lower, at 85.71%, and the Precision of external damage faults was 88.89%. The confusion matrix indicates that a small number of misclassifications still occurred among several environmentally induced or external-action fault categories, suggesting that some fault types exhibit overlapping transient traveling-wave patterns. Nevertheless, the proposed model still achieved an overall accuracy of 95.40% on real test samples, indicating that the GAF dual-branch image representation and PLO-selected explicit features can effectively improve the comprehensive discrimination capability for complex fault categories.

#### 5.2.3. Robustness Evaluation Based on Stratified Five-Fold Cross-Validation

The fixed stratified 8:2 hold-out test was used as the main experimental setting for detailed performance analysis and fair comparison among different model variants and baseline methods. To further verify the robustness of the proposed model under limited sample conditions, stratified five-fold cross-validation was additionally conducted using all 871 real historical fault samples. In each fold, approximately four fifths of the samples were used for model training and internal validation, while the remaining one fifth was used only as the independent test fold. To avoid information leakage, normalization-parameter estimation, Borderline-SMOTE, PLO-based feature selection, and model training were performed only using the training/validation part of each fold. The test fold was not involved in sample balancing, feature selection, hyperparameter tuning, or model training. The mean, standard deviation, and 95% confidence interval of the evaluation indicators were calculated across the five folds.

As shown in Table 5, the proposed model achieves an average Accuracy of 94.83 ± 0.91%, Recall of 95.54 ± 0.88%, Precision of 91.37 ± 1.07%, F1-score of 93.14 ± 0.81%, and AUC of 0.9947 ± 0.0019 across the five folds. The corresponding 95% confidence intervals are 94.83 ± 1.13% for Accuracy, 95.54 ± 1.10% for Recall, 91.37 ± 1.33% for Precision, 93.14 ± 1.01% for F1-score, and 0.9947 ± 0.0023 for AUC. Compared with the fixed hold-out test result, the five-fold cross-validation result remains at a similar performance level, indicating that the proposed model does not rely on a single favorable train/test split. The small standard deviations and narrow confidence intervals further demonstrate the robustness and statistical stability of the proposed model under limited and imbalanced historical fault samples.

For the subsequent comparative experiments, each experimental setting was first determined under the same representative stratified 8:2 train/test split. After the selected feature subset and model configuration were fixed for each setting, the corresponding model was independently trained 10 times with different random seeds. The reported indicators in the following comparison tables are the average values over the 10 independent training runs. This setting is used for the comparisons under different fault-category settings, different feature selection methods, different recognition models, and different ablation variants.

#### 5.2.4. Comparison of Identification Performance Under Different Numbers of Fault Categories

Table 6 presents the average identification results of the GAF-PCNN-AT model with PLO-selected features for 3-, 4-, 5-, and 6-class fault identification tasks over 10 independent training runs. As the number of fault categories increases, the model needs to perform discrimination under more complex class-boundary conditions. Therefore, its identification performance can reflect the adaptability of the proposed method under different classification granularities.

The 3-, 4-, and 5-class tasks are constructed by gradually adding fault categories from the six fault samples according to typicality and sample size. All tasks adopt the same data partition ratio and model parameter settings to ensure the comparability of results under different classification granularities.

As shown in Table 6, the average Accuracy of the model remains between 95.40% and 95.56% in the 3- to 6-class fault identification tasks. The macro-average Recall, Precision, and F1-score remain above 94%, and the average AUC remains between 0.9900 and 0.9941. As the number of fault categories increases, the complexity of class boundaries also increases. Compared with the 3-class task, the Accuracy, average Precision, average F1-score, and average AUC in the 6-class task decrease slightly, while the average Recall remains generally stable. Overall, the fluctuations in all indicators are small, indicating that the proposed model maintains stable identification performance under different classification granularities. In particular, under the 6-class condition, the model still achieves an Accuracy of 95.40% and an average AUC of 0.9900, demonstrating that the GAF-PCNN-AT model with PLO-selected features maintains high identification accuracy and good overall classification performance in more complex fault classification scenarios.

#### 5.2.5. Comparison of Different Feature Selection Methods

Table 7 presents the fault identification results of the model under different feature selection strategies. Each method uses GASF, GADF, and the corresponding explicit features as inputs, and the comparison is conducted under the same model structure and training conditions to verify the improvement effect of the PLO-based feature selection strategy on model identification performance.

As shown in Table 7, different feature selection strategies affect the utilization of key features under the same model structure. Without feature selection, all 42-dimensional explicit features are used as inputs. Although relatively complete information is retained, many redundant features are also included, resulting in relatively lower overall identification performance. After Lasso-based selection, all indicators are improved, indicating that appropriate dimensionality reduction can help reduce the interference caused by redundant features. The two swarm-intelligence-based selection methods, namely Genetic Algorithm (GA) and Binary Particle Swarm Optimization (BPSO), also improve model performance to some extent, but their overall performance remains lower than that of PLO. In contrast, the model using PLO-selected features achieves the highest Accuracy, average Recall, and average F1-score, reaching 95.40%, 95.68%, and 94.82%, respectively. Its average AUC reaches 0.9900, and its average Precision is 94.30%, which is close to the best Precision result. Compared with the methods without feature selection, Lasso, GA, and BPSO, the Accuracy of the PLO-selected model is improved by 1.55, 0.97, 1.26, and 1.49 percentage points, respectively. These results indicate that PLO can more effectively select key features closely related to fault class discrimination. By reducing input redundancy and enhancing the matching degree between the feature subset and fault patterns, PLO improves the overall identification performance of the model.

From the perspective of feature-subset search, Lasso is a filter/embedded feature selection method, whereas GA, BPSO, and PLO belong to population-based search methods. Compared with simple feature ranking methods, population-based methods can evaluate feature subsets as a whole and better consider the coupling relationship between selected features and the subsequent classifier.

Among them, PLO is adopted in this study because it provides a practical balance between global exploration and local exploitation for binary feature-subset search. As reported in Table 2 and Figure 8, the PLO-based feature selection process uses a population size of 20 and a maximum of 30 iterations, corresponding to an upper-bound evaluation budget of 600 function evaluations. The recorded computational cost under this complete iteration budget is within 101.22 s. The convergence curve shows that the best fitness value reaches a stable plateau within the given iteration range, indicating stable convergence before the full iteration budget is exhausted.

Since PLO-based feature selection is performed offline during model construction, this cost does not affect the online fault type identification stage. Therefore, PLO provides competitive recognition performance with stable convergence and acceptable offline computational cost in the proposed framework.

#### 5.2.6. Performance Comparison of Different Recognition Models

To further verify the effectiveness of the proposed model, commonly used machine learning and deep learning hybrid models in transmission line fault classification and localization are selected as comparison methods with reference to existing studies [20]. The proposed GAF-PCNN-AT model with PLO-selected features is compared with CNN-SVM, CNN-BiLSTM-Attention, and GAF-PCNN. For CNN-SVM, CNN is first used to extract dual-branch GAF image features, and the extracted high-level image features are then fed into a Support Vector Machine (SVM) classifier for fault identification. CNN-BiLSTM-Attention further introduces Bidirectional Long Short-Term Memory (BiLSTM) and an attention mechanism on the basis of convolutional feature extraction to enhance feature modeling capability. GAF-PCNN is used to verify the effectiveness of dual-branch GAF image representation in the fault identification task. The performance comparison results of different recognition models are presented in Table 8.

**Figure 11:**
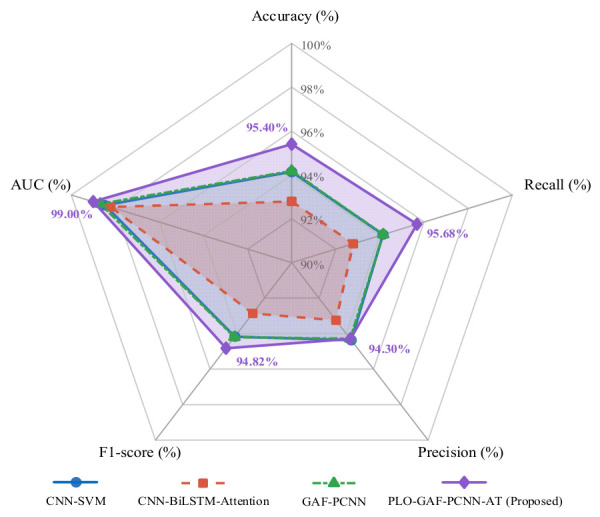
Multi-metric radar chart of different recognition models.

To more intuitively show the comprehensive differences among different recognition models in terms of Accuracy, Recall, Precision, F1-score, and average AUC, a multi-metric radar chart is further plotted, as shown in Figure 11.

As shown in Table 8, the proposed GAF-PCNN-AT model with PLO-selected features achieves the best overall performance. Its Accuracy reaches 95.40%, and its average AUC reaches 0.9900, both of which are higher than those of CNN-SVM, CNN-BiLSTM-Attention, and GAF-PCNN. Compared with these three baseline models, the proposed method improves the Accuracy by 1.26, 2.64, and 1.20 percentage points, respectively. Although the average Precision of CNN-SVM is slightly higher than that of the proposed model, the proposed model obtains better results in Accuracy, average Recall, average F1-score, and average AUC. These results indicate that a single classifier or a single-path image model has difficulty simultaneously considering the global correlation structure, local difference pattern, and multi-domain physical-feature information of traveling-wave signals. In contrast, the proposed method achieves complementary enhancement among different representations through GASF/GADF dual-branch image representation, PLO-selected multi-domain explicit features, and a multi-head attention fusion mechanism, thereby obtaining better overall identification performance.

#### 5.2.7. Ablation Study of the Three-Branch Fusion Architecture

To further verify the effectiveness of the PLO-selected explicit feature branch and the multi-head attention module, an ablation study was conducted by comparing four model variants. The results are shown in Table 9.

As shown in Table 9, adding the multi-head attention module to GAF-PCNN improves Accuracy and Recall, but Precision and F1-score decrease when explicit physical features are not introduced. This indicates that attention applied only to image features may enhance useful responses while also amplifying some ambiguous image patterns. Introducing the PLO-selected explicit feature branch improves Precision and F1-score compared with GAF-PCNN-AT, showing that selected physical features provide complementary discriminative information. The complete proposed model achieves the best overall performance, indicating that the PLO-selected explicit feature branch and the multi-head attention module work more effectively when used jointly. These results verify the necessity of the three-branch fusion design.

#### 5.2.8. Qualitative Comparison with Recent GAF-Based Fault Diagnosis Methods

To further clarify the methodological difference between the proposed framework and recent GAF-based fault diagnosis methods, a qualitative comparison is provided in Table 10. Since the related studies differ in data sources, signal types, fault categories, sampling conditions, and evaluation protocols, a direct numerical comparison of accuracy may not be fully fair. Therefore, this subsection focuses on representation construction, feature utilization, and fusion architecture.

As shown in Table 10, recent GAF-based fault diagnosis methods mainly improve fault recognition from the perspectives of image transformation, classifier-backbone design, attention enhancement, or multi-channel image-feature extraction. These studies demonstrate the effectiveness of GAF-type images for transforming one-dimensional non-stationary signals into two-dimensional correlation representations. However, for transmission line traveling-wave signals, some physically meaningful information may be weakened during normalization, image transformation, and image resizing. For example, the PLO-selected explicit features in this study, such as peak-to-peak value, root mean square, root mean square frequency, wavelet packet energy ratio, wavelet singular entropy, pulse width, and rise time, provide complementary descriptions of amplitude variation, frequency distribution, time–frequency characteristics, and waveform-edge morphology.

Therefore, the explicit feature branch complements GAF images by providing compact physical descriptors selected from the original 42-dimensional candidate feature set. GASF is used to describe the global correlation structure of the traveling-wave sequence, and GADF is used to enhance local difference patterns and transient variations. Since directly using all explicit features may introduce redundant or weakly relevant information, PLO is used to select compact discriminative features before fusion. Compared with filter-based feature ranking, PLO-based feature selection can be formulated as a binary subset search process guided by validation classification performance. This makes the selected feature subset more consistent with the subsequent GAF-PCNN-AT fusion model.

In addition, the comparison with Lasso, GA, and BPSO-based feature selection methods, together with the reported PLO convergence curve and computational cost, further supports the use of PLO in the proposed framework.

The above comparison shows that the proposed method constructs a feature-optimized heterogeneous representation framework for imbalanced transmission line fault type identification. By combining independent GASF/GADF image learning, PLO-selected explicit physical features, and multi-head attention fusion, the proposed framework jointly improves representation completeness, feature compactness, and fusion adaptability. These observations further suggest that image-only GAF representation is effective in learning angular-correlation structures, but it may still be insufficient for fault categories with similar transient waveform morphology. Optimized multi-domain physical features can supplement deep GAF image features and help improve the stability and interpretability of fault type identification.

## 6. Discussion

The experimental results show that the proposed GAF-PCNN-AT model with PLO-selected features can effectively identify six types of transmission line faults using historical traveling-wave data acquired from distributed online monitoring devices. The six-class Accuracy of 95.40% and the average AUC of 0.9900 indicate that the proposed method maintains good classification performance under real imbalanced fault sample conditions. This supports the assumption that fault traveling-wave signals can be more effectively characterized by combining image-based deep representations with physically interpretable explicit features, rather than relying on a single feature form. The stratified five-fold cross-validation results further support this observation, with an average Accuracy of 94.83 ± 0.91% and an average AUC of 0.9947 ± 0.0019 across folds, indicating that the reported performance does not rely on a single favorable train/test split.

The results under different numbers of fault categories further demonstrate the adaptability of the proposed framework. As the number of fault categories increases from three to six, the class boundaries become more complex, but the Accuracy remains within a narrow range of 95.40–95.56%, and the average AUC remains within 0.9900–0.9941. This suggests that the GASF/GADF dual-branch representation and PLO-selected multi-domain explicit features provide complementary information for different classification granularities. Specifically, GASF captures the global correlation structure of traveling-wave sequences, whereas GADF enhances local differences and waveform-edge variations. This complementary representation is beneficial for distinguishing fault categories with similar transient waveform patterns.

The comparisons with different feature selection strategies and recognition models further verify the necessity of the proposed design. PLO selects an 18-dimensional feature subset from the original 42-dimensional explicit features, reducing redundant and weakly relevant inputs while retaining features closely related to fault discrimination. Compared with no feature selection, Lasso, GA, and BPSO, the PLO-selected model achieves the best overall Accuracy and F1-score. In addition, compared with CNN-SVM, CNN-BiLSTM-Attention, and GAF-PCNN, the proposed model achieves higher Accuracy, average Recall, average F1-score, and average AUC. These results indicate that the combination of dual-branch GAF image representation, PLO-selected explicit features, and multi-head attention fusion can better integrate global correlation information, local difference information, and multi-domain physical features. The ablation results further confirm this interpretation, showing that the PLO-selected explicit feature branch and the multi-head attention module are most effective when used jointly in the complete three-branch fusion architecture.

Some misclassifications still occur among several environmentally induced or external-action fault categories. This is reasonable because these environmental or external-action faults may exhibit partially overlapping transient traveling-wave characteristics, especially in terms of wavefront abruptness, local oscillation, and high-frequency attenuation. Therefore, the remaining errors reflect not only model limitations but also the intrinsic similarity of certain real fault waveforms. In future work, the dataset will be expanded across different regions, voltage levels, and time spans. Additional field information, such as meteorological records, line operating conditions, and multi-terminal synchronized measurements, may also be introduced to improve the discrimination of confusing fault categories. Moreover, lightweight model design and real-time deployment should be further investigated for online transmission line monitoring applications.

## 7. Conclusions

This study proposed a transmission line fault type identification method based on PLO-selected features and a GAF-PCNN-AT fusion network. The method integrates Borderline-SMOTE-based training sample balancing, multi-domain explicit feature extraction, GASF/GADF dual-branch image representation, PLO-based feature selection, and multi-head attention fusion. Through this design, the proposed framework jointly exploits the global correlation structure, local difference pattern, and physically interpretable multi-domain explicit features of fault traveling-wave signals.

Validation on the independent real test set under a representative stratified 8:2 split shows that the proposed method achieves an Accuracy of 95.40% and an average AUC of 0.9900 in the six-class fault identification task. The comparison results indicate that PLO-selected features can reduce redundant explicit inputs, while the GAF-PCNN-AT model can adaptively fuse dual-branch image features and selected explicit features to improve overall identification performance. The proposed method provides an effective intelligent diagnostic approach for transmission line fault type identification using traveling-wave data recorded by online monitoring devices.

## Figures and Tables

**Table 1:** Selected 18-dimensional explicit feature subset obtained by PLO from the 42-dimensional candidate feature set.

Feature Category	Selected Features	
Time domain features	Mean value	Peak-to-peak value
	Square-root amplitude	Root mean square
	Skewness	Waveform factor
	Crest factor	Impulse factor
	Peak factor	
Frequency domain features	Mean frequency	Root mean square frequency
Time–frequency domain features	Wavelet packet energy ratio (sub-bands 5 and 7)	Wavelet packet scale entropy (sub-bands 2 and 4)
	Wavelet singular entropy (all eight terminal sub-bands)	
Waveform-edge features	Pulse width	Rise time

**Table 2:** Parameter settings and computational cost of the PLO-based feature selection process.

Parameter	Value/Setting
Population size	20
Maximum iterations	30
Maximum function evaluations	600
Candidate feature dimension	42
Selected feature dimension	18
Initialization strategy	Random initialization within search boundaries [0, 1]
Fitness function	Validation accuracy with feature-size penalty, as defined in Equation (7)
Computational cost per run	101.22 s

**Table 3:** Model structure parameters and training hyperparameters of the proposed GAF-PCNN-AT model with PLO-selected features.

Parameter	Value
Input image size	227 × 227 × 3
Dimension of PLO-selected features	18
Convolution kernel size/number	7 × 7/64
Pooling kernel size/stride	3 × 3/2
Dimension of the fusion fully connected layer	128
Number of multi-head attention heads	4
Mini-batch size	16
Initial learning rate	8 × 10^−5^
Maximum number of training epochs	120
L2 regularization coefficient	1 × 10^−4^
Optimizer	Adam
Learning rate schedule	Fixed
Loss function	Cross-entropy loss
Data shuffling strategy	Every epoch
Software platform	MATLAB R2024a
Hardware environment	Intel Core i5-12400F CPU, NVIDIA GeForce RTX 3060 GPU

**Table 4:** Category-wise evaluation indicators of the proposed model under a representative stratified 8:2 train/test split.

Fault Type	Evaluation Indicator				
	Recall/%	Precision/%	F1-Score/%	AUC	Overall Accuracy/%
Icing	85.71	92.31	88.89	0.9824	95.40
Wind deviation	94.12	100.00	96.97	0.9753	
External damage	96.97	88.89	92.75	0.9845	
Bird-related faults	100.00	91.67	95.65	1.0000	
Other faults	100.00	91.67	95.65	1.0000	
Lightning strikes	97.30	100.00	98.63	0.9980	

**Table 5:** Stratified five-fold cross-validation results of the proposed GAF-PCNN-AT model with PLO-selected features on the whole 871-sample dataset.

Fold	Accuracy/%	Recall/%	Precision/%	F1-Score/%	AUC
Fold 1	95.40	95.68	92.41	93.87	0.9967
Fold 2	94.83	96.21	91.14	93.21	0.9944
Fold 3	94.25	94.11	90.96	92.33	0.9950
Fold 4	95.98	96.29	92.42	94.00	0.9956
Fold 5	93.68	95.39	89.90	92.30	0.9917
Mean ± Std	94.83 ± 0.91	95.54 ± 0.88	91.37 ± 1.07	93.14 ± 0.81	0.9947 ± 0.0019
95% CI	94.83 ± 1.13	95.54 ± 1.10	91.37 ± 1.33	93.14 ± 1.01	0.9947 ± 0.0023

**Table 6:** Average recognition performance of the proposed GAF-PCNN-AT model with PLO-selected features under different fault-category settings over 10 independent training runs.

Number of Fault Categories	Accuracy/%	Average Recall/%	Average Precision/%	Average F1-Score/%	Average AUC
3	95.56	95.69	95.71	95.61	0.9941
4	95.53	95.50	95.62	95.54	0.9938
5	95.52	95.58	95.66	95.54	0.9939
6	95.40	95.68	94.30	94.82	0.9900

**Table 7:** Average recognition performance comparison of different feature selection methods under the same GAF-PCNN-AT model structure over 10 independent training runs.

Feature Selection Method	Accuracy/%	Average Recall/%	Average Precision/%	Average F1-Score/%	Average AUC
Without feature selection	93.85	93.82	93.95	93.88	0.9887
Lasso selection	94.43	94.38	94.49	94.43	0.9902
GA selection	94.14	94.14	94.25	94.14	0.9895
BPSO selection	93.91	93.89	94.03	93.94	0.9890
PLO selection (proposed)	95.40	95.68	94.30	94.82	0.9900

**Table 8:** Average recognition performance comparison of different recognition models for six-class transmission line fault identification over 10 independent training runs.

Recognition Model	Accuracy/%	Average Recall/%	Average Precision/%	Average F1-Score/%	Average AUC
CNN-SVM	94.14	94.14	94.37	94.17	0.9847
CNN-BiLSTM-Attention	92.76	92.79	93.25	92.86	0.9821
GAF-PCNN	94.20	94.15	94.29	94.20	0.9868
GAF-PCNN-AT with PLO-selected features (proposed)	95.40	95.68	94.30	94.82	0.9900

**Table 9:** Ablation results of the PLO-selected explicit feature branch and multi-head attention module over 10 independent training runs.

Model Variant	PLO-Selected Explicit Feature Branch	Multi-Head Attention	Accuracy/%	Recall/%	Precision/%	F1-Score/%	AUC
GAF-PCNN	×	×	94.20	94.15	94.29	94.20	0.9868
GAF-PCNN-AT	×	√	94.54	95.25	91.20	92.76	0.9844
GAF-PCNN+PLO features	√	×	94.43	94.87	93.42	93.92	0.9883
Proposed GAF-PCNN-AT	√	√	95.40	95.68	94.30	94.82	0.9900

Note: “√” denotes that the corresponding module is used in the model variant, whereas “×” denotes that the corresponding module is not used.

**Table 10:** Qualitative comparison between recent GAF-based fault diagnosis methods and the proposed method.

Method Category	Representative Studies	Main Methodological Focus	Relative Advantage of the Proposed Method
GAF with advanced classifiers	GAF-CNN-ViT [21]; GAF-optimized ShuffleNetV2 [18]	Classifier-backbone enhancement	Heterogeneous GASF/GADF/explicit-feature inputs
Single GAF/GADF with attention or residual learning	GADF-IDARN [22]	Single-image representation enhancement	Complementary GASF/GADF branch learning
GAF with parallel or dual-channel CNNs	GASF-parallel CNN [23]; GAF-DC-CNN [24]	Multi-branch image-feature extraction	Heterogeneous image-feature and physical-feature fusion
Proposed GAF-PCNN-AT	This study	GASF/GADF+PLO-selected explicit features+attention fusion	Representation completeness, feature compactness, and adaptive fusion

## Data Availability

The datasets presented in this article are not readily available because the raw traveling-wave fault records contain confidential field operation and fault information from actual transmission lines. Requests to access additional datasets should be directed to the corresponding author and are subject to permission from the data provider.

## Abbreviations

The following abbreviations are used in this manuscript:

AUC	Area Under the Curve
BiLSTM	Bidirectional Long Short-Term Memory
Borderline-SMOTE	Borderline Synthetic Minority Over-sampling Technique
BPSO	Binary Particle Swarm Optimization
CNN	Convolutional Neural Network
CWT	Continuous Wavelet Transform
FFT	Fast Fourier Transform
GADF	Gramian Angular Difference Field
GAF	Gramian Angular Field
GAF-PCNN-AT	Gramian Angular Field-Parallel Convolutional Neural Network-Attention
GASF	Gramian Angular Summation Field
GA	Genetic Algorithm
HVDC	High-Voltage Direct Current
PCNN	Parallel Convolutional Neural Network
PLO	Polar Lights Optimizer
ReLU	Rectified Linear Unit
ROC	Receiver Operating Characteristic
SMOTE	Synthetic Minority Over-sampling Technique
SVM	Support Vector Machine
WPD	Wavelet Packet Decomposition
WPT	Wavelet Packet Transform
